# Carta al editor “Tuberculosis multirresistente en Colombia, 2013-2018: estudio de casos y controles”

**DOI:** 10.7705/biomedica.7553

**Published:** 2024-05-30

Lima, 19 de marzo de 2024

Señores Comité Editorial Revista *Biomédica*

Estimados señores:

Hemos leído con gran interés el artículo de Puerto *et al*. “Tuberculosis multirresistente en Colombia, 2013-2018: estudio de casos y controles”, publicado en la revista *Biomédica* Volumen 43, N°4, diciembre de 2023. El estudio presenta un análisis de patrones de comportamiento y factores de riesgo, fundamental para desarrollar estrategias efectivas de prevención y tratamiento. Los hallazgos refuerzan la importancia de abordar las desigualdades en el acceso a la atención médica y el fortalecimiento de los sistemas de salud al ofrecer evidencia sólida que fortalece políticas y programas de salud pública.

Coincidimos con los autores en que la identificación oportuna de los casos de TB MDR es fundamental para el control de la enfermedad, siendo necesario fortalecer los sistemas de vigilancia y diagnóstico, así como mejorar el acceso al tratamiento. Sin embargo, algunas diferencias importantes entre los casos notificados y los casos estimados podrían tener implicancias para la interpretación de los resultados y la formulación de políticas.

Resalta que no cruzaron los datos obtenidos con la información demográfica de la población en regiones clave como Antioquia, Valle del Cauca, Risaralda y Bogotá, paso crucial para comprender el impacto y la distribución de la TB MDR en áreas densamente pobladas e imposibilita la comparación con otras regiones, limitando la utilidad del estudio para comprender la dinámica de la TB MDR.

La diversidad étnica y cultural colombiana es una característica distintiva que refleja desafíos en términos de acceso a la atención médica y exposición a factores de riesgo de enfermedades ^2^. En el estudio, el 80% de los pacientes fueron clasificados en el rubro de “otros” ignorando la etnicidad lo que impide al estudio comprender la asociación con la TB MDR y plantea cuestiones que merecieron una mayor exploración y discusión en el estudio, como comparar las tasas de TB MDR y el riesgo en las etnias, el análisis de las diferencias debido a niveles de acceso a la atención y necesidades en términos de prevención y control de la tuberculosis.

El análisis presentado muestra que el 15% de los casos y los controles están en la categoría de sobrepeso u obesidad; este hallazgo plantea consideraciones sobre la interacción entre la tuberculosis multirresistente y el estado nutricional de la población cuya relación ha sido estudiada ^3^. El sobrepeso aumenta el riesgo de tuberculosis e influye en la progresión y la respuesta al tratamiento de la enfermedad. En el contexto colombiano con ambos problemas de salud pública, entender la interrelación es esencial para diseñar estrategias efectivas de prevención y tratamiento. Por ello, un análisis detallado de esta asociación que incluya la comparación de las tasas de TB MDR entre personas con peso normal, sobrepeso y obesidad, análisis de posibles razones de las diferencias en las tasas de TB MDR en las diferentes categorías de peso; asociación entre el sobrepeso y obesidad con mayor riesgo de desarrollar TB MDR o mayor gravedad de la enfermedad hubiese ayudado a comprender la existencia de relaciones de causalidad entre ellas.

El resultado de un *odds ratio* (OR) de 907.96 para la presencia de, al menos, una comorbilidad llama la atención por ser alto e indicar asociación muy fuerte entre la presencia de, al menos, una comorbilidad y la TB MDR, dato ignorado en los comentarios, existiendo varias posibles explicaciones:

- las personas con comorbilidades tienen un sistema inmunológico comprometido o debilitado que las hace más susceptibles a la infección por tuberculosis y a desarrollar formas multirresistentes;
- algunas comorbilidades tienen factores de riesgo compartidos asociados con un mayor riesgo de desarrollar tuberculosis por el impacto en el sistema inmunológico, predisponiendo a formas más graves o multirresistentes de la enfermedad;
- dificultades en el manejo de múltiples condiciones de salud;
- interacciones farmacológicas;
- el tamaño de la muestra puede influir en el tamaño del OR, puede verse afectada con una mayor variabilidad en los datos cuando se trabaja con muestras pequeñas o poblaciones limitadas ^4^.

Finalmente, el análisis de las tasas de desnutrición, diabetes, VIH y farmacodependencia, la distribución racial y étnica, la cobertura del programa de prevención y control de la tuberculosis en las cuatro regiones comparado con el resto del país, además de robustecer los resultados, proporcionaría información valiosa para el diseño de estrategias de prevención y control de la TB MDR.

César Antonio Bonilla-Asalde

DNI: 16498481

Isabel Cristina Rivera-Lozada

DNI: 66767305

Oriana Rivera-Lozada

DNI: 48664887
