# Supplementary material for: Carta al editor “Tuberculosis multirresistente en Colombia, 2013-2018: estudio de casos y controles”
Source: Biomedica. 2024 May 30;44(2):277–80. [Article in Spanish] doi: 10.7705/biomedica.7553 (PMC11309784; doi:10.7705/biomedica.7553)
Supplement: Related texts

# Reply: Cartas al Editor

Bogotá, 5 de abril de 2024

Doctor

Cesar Antonio Bonilla

Lima, Perú

Estimado doctor:

Reciba un cordial saludo.

Agradecemos su interés en el artículo “Tuberculosis multirresistente en Colombia, 2013-2018: estudio de casos y controles”, publicado en la revista Biomédica, volumen 43, No. 4, diciembre de 2023.

Los datos analizados en el manuscrito corresponden única y exclusivamente a la información disponible y real para Colombia de los casos de TB MDR, recopilados por el Programa Nacional de Control de Tuberculosis entre los años 2013 y 2018. Se trabajó con la totalidad de los datos existentes, recolectados en el marco de la vigilancia de la tuberculosis; estos hallazgos reflejan la realidad de los casos de TB MDR bajo los parámetros establecidos por el Programa Nacional en el país en este periodo, limitándonos al análisis de datos procedentes de fuentes secundarias.

Los resultados muestran que más del 50 % de los casos TB MDR se concentraron en Antioquia y Valle del Cauca seguido de los casos TB MDR presentados en Risaralda y Bogotá. No ocurrieron casos de TB MDR en otros departamentos, por lo que no es posible hacer comparaciones de tasas de incidencia con los incluidos en este estudio. Las variables disponibles fueron analizadas.

Con respecto al análisis de etnias, sucede lo mismo. La categoría “otros” es la existente en el sistema de vigilancia epidemiológica del país; esta categoría está conformada por todas las personas que no pertenecen a las demás etnias contempladas por el sistema de vigilancia epidemiológica del país (https://www.ins.gov.co/buscador-eventos/Lineamientos/Datos_B%C3%A1sicos_2024.pdf) categorización establecida por el Departamento Administrativo Nacional de Estadística (DANE).

Por otra parte, la variable cobertura del programa no se encuentra documentada en las bases de datos secundarias, y el levantamiento de esa información requiere trabajo de campo, el cual no formó parte de este estudio y para lo cual es necesario, de un lado, la consecución de recursos y, de otro lado, la creación del campo para la variable cobertura en el sistema de información, con dificultades de recolección retrospectiva. En todo caso, la información de la variable cobertura para el periodo 2013-2018 no existe para el país.

Para la variable “peso”, debido al bajo número de pacientes que aparecían en la base con sobrepeso y obesidad, se decidió conformar una nueva variable con las categorías descritas en los análisis de regresión. Por la misma razón, las variables “desnutrición”, “diabetes” y “farmacodependencia” se agruparon en una nueva variable llamada “factores de riesgo” con las categorías “sí”: presencia de al menos un riesgo (desnutrición, diabetes o farmacodependencia) y la categoría “no”: ausencia de riesgos. Este reagrupamiento de las categorías de las variables se realizó para que los datos fueran consistentes para correr la prueba de regresión y correlación.

Muchas gracias por las observaciones, serán tenidas en cuenta para el desarrollo de estudios futuros de TB-MDR.

Atentamente,

Gloria Mercedes Puerto Castro
